# Genetic Analysis of the Complete S1 Gene in Japanese Infectious Bronchitis Virus Strains

**DOI:** 10.3390/v14040716

**Published:** 2022-03-29

**Authors:** Masaji Mase, Kanae Hiramatsu, Satoko Watanabe, Hiroshi Iseki

**Affiliations:** 1 National Institute of Animal Health, National Agriculture and Food Research Organization, 3-1-5 Kannondai, Tsukuba 305-0856, Japan; satochan@affrc.go.jp (S.W.); hiseki@affrc.go.jp (H.I.); 2 United Graduate School of Veterinary Sciences, Gifu University, 1-1 Yanagido, Gifu 501-1193, Japan; 3 Graduate School of Life and Environmental Sciences, Osaka Prefecture University, Izumisano 598-8531, Japan; 4 Oita Livestock Hygiene Service Center of Oita Prefecture, 442 Onozuru, Oita 870-1153, Japan; hiramatsu-kanae@pref.oita.lg.jp

**Keywords:** genotype, infectious bronchitis virus, phylogeny, S1 gene

## Abstract

The complete nucleotide sequence of the S1 glycoprotein gene of the Japanese infectious bronchitis virus (IBV) strains was determined and genetically analyzed. A total of 61 Japanese IBV strains were classified into seven genotypes, namely GI-1, 3, 7, 13, 18, 19, and GVI-1 using the classification scheme that was proposed by Valastro et al, with three exceptions. These genotypes practically corresponded to those defined in Japan, namely Mass, Gray, JP-II, 4/91, JP-I, JP-III, and JP-IV, which have been identified through their partial nucleotide sequences containing hypervariable regions 1 and 2. In addition, three exceptive strains were considered to be derived from recombination within the S1 gene of IBV strains G1-13 and GI-19. By analyzing the amino acid polymorphism of the S1 glycoprotein among Japanese genotypes, a diversity was observed based on the genotype-specific amino acid residue, the proteolytic cleavage motif at the S1/S2 cleavage site, and the position of the potential N-glycosylation sites.

## 1. Introduction

Avian infectious bronchitis virus (IBV) causes a highly contagious respiratory, and sometimes urogenital, disease that affects egg production and shell quality in layer chickens [1]. Mortality due to IBV infection alone is usually very low, but it can be significant following secondary infections with bacteria such as *Escherichia coli*. Therefore, IBV infection is considered the second most damaging poultry disease worldwide after highly pathogenic influenza [2]. To protect poultry from it, live or inactivated vaccines are used [3]. However, the protection that is conferred by vaccination is incomplete because the high mutation frequency of IBV leads to the emergence of new strains [1].

The causative coronavirus is an enveloped and positive-stranded RNA virus, containing an unsegmented genome of approximately 27.6 kb. IBV has three major virus-encoded structural proteins: the spike (S) glycoprotein, membrane (M) protein, and nucleocapsid (N) protein. The IBV spike is formed by the post-translational cleavage of S1 and S2 polypeptides [4]. Among these spike components, the S1 glycoprotein is associated with viral attachment and is a primary target of neutralizing antibodies in chickens [5,6]. Therefore, an analysis of the S1 gene is important to characterize the isolated IBVs.

Consequently, based on the nucleotide sequence of the S1 region on the spike gene (S1 gene), the genetic grouping of IBV has mainly been conducted [7,8,9]. As a result, prevalent nucleotide diversity was revealed in this region containing three hypervariable regions (HVRs) (aa 38–67, 91–141, and 274–387). Moreover, using complete nucleotide sequences of the S1 gene, IBV strains recently distributed worldwide between 1937 and 2013 have been classified into 32 phylogenetic lineages (GI-1 to GI-27 and GII to GVI) [10]. In contrast, IBV strains in Japan have been genotyped using partial nucleotide sequences containing the HVR-1 and 2 regions [11,12]. Based on this classification, they were classified into seven genotypes: the JP-I, J-II, JP-III, JP-IV, Mass, 4/91, and Gray. However, it remains unknown if this classification system that was adopted in Japan corresponds to that which was described in Valastro et al. [10] since the complete S1 gene has been determined only for a limited number of IBV strains in Japan.

Furthermore, the cryo-electron microscopy structure of the M41 strain spike has recently been resolved [13]. Therefore, to understand the infectivity and pathogenicity of viruses, it would be imperative to examine the amino acids that are related to the receptor-biding domain (RBD) of the virus. This study indicated that the S1 subunit comprised of two independent folding domains: the N-terminal domain (NTD) (amino acids 21 to 237) and the C-terminal domain (amino acids 269 to 414), with a proposed receptor-binding site at both domains. Additionally, the glycosylation of spike proteins influences many of the processes and outcomes of an IBV infection, including the antigenicity, infectivity, and receptor-binding [14]. Therefore, comparing N-glycosylation sites among IBV strains is also essential to examine such biological functions. However, little analysis on the structure of such S1 glycoproteins, including the amino acids that are related to the RBD or potential N-glycosylation sites, have been performed on Japanese IBV strains.

This study determined the complete S1 gene nucleotide sequences of 61 IBV Japanese strains. Then, their genotype definitions were compared with those that were reported in the classification by Valastro et al. [10]. Additionally, amino acid polymorphisms in the S1 glycoproteins, such as the RBD, the motif at the S1/S2 cleavage site, and the position of the potential N-glycosylation site with reference strains, were analyzed among Japanese genotypes.

## 2. Materials and Methods

### 2.1. Viruses

The IBV strains that were examined in this study are shown in Table 1. The allantoic cavities of embryonated eggs were used for virus propagation. After inoculation, the eggs were incubated at 37 °C for 3–4 days unless the embryo died. The inoculated eggs were then chilled to 4 °C, and the allantoic fluids were harvested.

### 2.2. RNA Extraction, RT-PCR, and Sequencing

Viral ribonucleic acid (RNA) was extracted from the infected allantoic fluids using a commercial kit (QIAamp Viral RNA Mini Kit; QIAGEN, Valencia, CA, USA) according to the manufacturer’s instructions. The primer sequences that were used for polymerase chain reaction (PCR) are shown in Table 2. These primers were designed to amplify the complete S1 gene and were used for detection and sequencing. After reverse transcription with Superscript IIi (Life Technologies, Gaithersburg, MD) using with random 9-mer oligonucleotides at 25 °C for 10 min and 42 °C for 60 min, cDNAs were amplified by PCR. PCR was performed at the following conditions: 35 cycles at 94 °C for 30 s, 50 °C for 30 s, and 72 °C for 30 s.

Each PCR product was purified with Montage (Millipore, Billerica, MA, USA) according to the manufacturer’s instructions. The purified PCR products were used as a template for sequencing on an Applied Biosystems 3130xl Genetic Analyzer using dye terminator cycle sequencing chemistry (Big Dye; Applied Biosystems, Foster City, CA, USA). The purified PCR products were sequenced from both directions.

### 2.3. Genetic Analysis of the S1 Gene

The sequenced fragments were assembled using ATGC-Mac ver.5 (GENETYX CORPORATION, Tokyo, Japan). The derived nucleotide sequences and deduced amino acids were then analyzed using GENETYX-Mac ver. 18.0 (GENETYX CORPORATION). The detection of potential N-glycosylation sites were also predicted using this software. The nucleotide sequences of the complete S1 glycoprotein-coding region were used to construct a phylogenetic tree in MEGA7 [15]. All tools were run with default parameters unless otherwise specified. All of the sequences that were obtained in this study were deposited in the GenBank database (accession numbers LC662545-LC662604).

### 2.4. Phylogenetic Analysis

**Figure 1:**
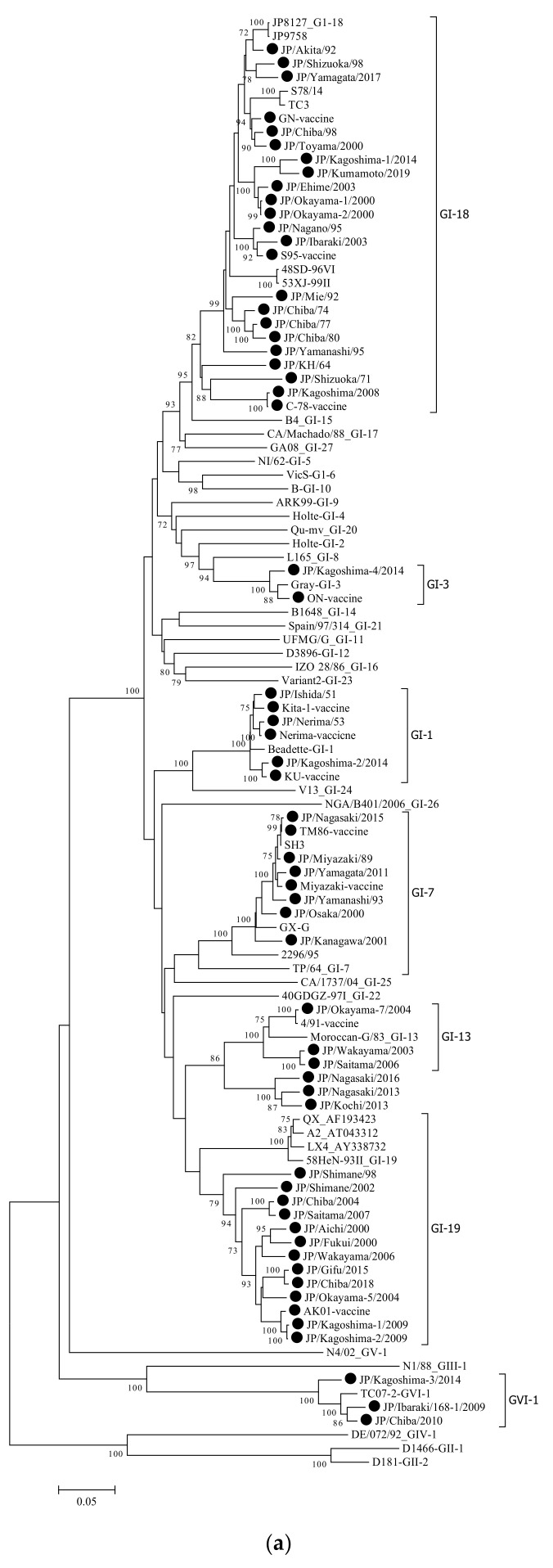

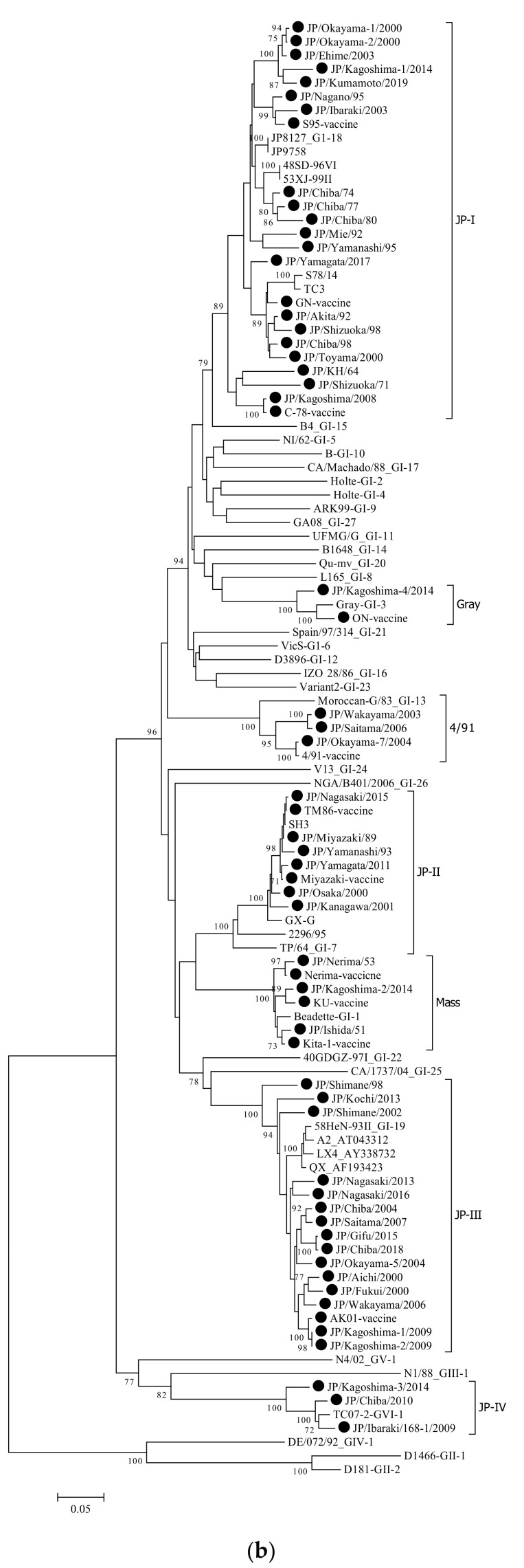
Phylogenetic trees that are based on the complete (**a**) and partial (**b**) S1 glycoprotein gene of the infectious bronchitis virus (IBV), strain Beaudette (GI-1, GenBank Accession No. NC001451). For (**a**,**b**), nucleotides 20368–21978 (1632 bases) and nucleotides 20368–20988 (621 bases), respectively, were subjected to phylogenetic analysis. Subsequently, both trees were generated using the neighbor-joining method in MEGA 7 [15] with 1000 bootstrap replications. All tools were run with the default parameters unless otherwise specified. Then, horizontal distances were proportionally set to the minimum number of nucleotide differences that were required to join nodes and sequences. The IBV genotypes were defined as described previously [10].

As previously described, phylogenetic trees were constructed using the neighbor-joining method [12]. Subsequently, to compare the Japanese genotypes with those that were reported in Valastro et al. [10], phylogenetic trees were constructed using two portions of the S1 gene (approximately from nt 1 to 1630 and from nt 1 to 600) (Figure 1a,b).

### 2.5. Recombination Analysis

Potential recombination events were further verified in SimPlot (version 3.5.1.) [16,17], after which nucleotide identity was determined using the Kimura (2-parameter) method with a transition–transversion ratio of 2 and a window width/step size of 200 bp and 20 bp, respectively. Subsequently, BootScan analysis was conducted, employing a subprogram embedded in SimPlot, in addition to signals of 70% or more of the observed permuted trees to indicate potential recombination events [16,17].

## 3. Results

### 3.1. Phylogenetic Analysis

DNA fragments of the expected size were successfully amplified from all IBV samples. The complete nucleotide sequences of the S1 glycoprotein gene were generated from the sequences of two overlapping PCR products. To follow the classification of Valastro et al., the complete length of the S1 gene was analyzed, whereas a partial analysis of the S1 gene was conducted to follow the Japanese classification. To determine the genetic relationships among the Japanese strains and foreign reference ones, a phylogenetic tree was constructed using the nucleotide sequences of the complete S1 gene of the 61 Japanese and 45 reference strains (Figure 1a,b).

The Japanese IBV strains were classified into seven distinct genetic groups (Figure 1a). These groups corresponded to clades GI-1,3,7,13,18,19, and GVI-1, as defined in previous report [10]. Subsequently, as shown in Figure 1b, they were also classified into seven groups by the Japanese definition based on the partial nucleotide sequences, including the HVR-1 and HVR-2 [11,12]. Except for three strains, GI-1,3,7,13,18,19, and GVI-1, corresponded to Mass, Gray, JP-II, 4/91, JP-I, JP-III, and JP-VI, respectively (Table 1). The exceptions were the three IBV strains that were classified as JP-III in Japan based on partial sequences of the S1 gene. Nevertheless, they were not classified as GI-19 when using the complete S1 gene. This result proposes that these strains were possibly recombinant, thus affecting the above classification.

### 3.2. Recombination Analysis

**Figure 2:**
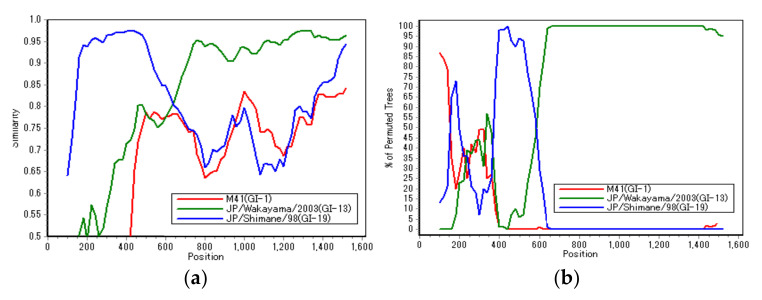
Similarity analysis (**a**) and BootScan analysis (**b**) on the putative recombinant JP/Nagasaki/2013 strain. Reference strains JP/Wakayama/2003 (GI-13, green) and JP/Shimane/98 (GI-19, blue) strains were used as putative parental strains. Additionally, the M41 strain (G1-1, red) was used as an outlier sequence. The *y*-axis indicates the percentage of identity within a 200-bp wide sliding window centered on the plotted position, with a step size of 20 bp between plots.

For three strains (JP/Nagasaki/2013, JP/Kochi/2013, and JP/Nagasaki/2016), in which the classification by Valastro et al. [10], and the Japanese one did not correspond (as shown in Table 1), a Simplot and BootScan analysis was conducted to verify the possibility of genetic recombination. These analyses were conducted to identify the recombinant JP/Nagasaki/2013 and reference JP/Wakayama/2003 (GI-13) and JP/Shimane/98 (GI-19) strains to be used as putative parental strains (Figure 2a,b).

As shown in Figure 2, the JP/Nagasaki/2013 strain was genetically close to the JP/Shimane/98 strain, belonging to the GI-19 (JP-III) genotype up to around 600 bp on the N-terminal side; however, the remaining on the C-terminal side were close to JP/Wakayama/2003, belonging to the GI-13 (4/91) genotype. Hence, in the S1 gene of these strains (JP/Nagasaki/2013, JP/Kochi/2013, and JP/Nagasaki/2016), approximately 600 bp on the N-terminal side were derived from the GI-19 genotype. However, the remaining region on the C-terminal side was derived from the GI-13 genotype. This result is consistent with that of a previous analysis [10], which showed that most of the recombination breakpoints of field IBV strains occur in the intermediate region between HVR1, HVR2, and HVR3.

### 3.3. Proteolytic Cleavage Sites in the Spike Glycoprotein

First, to examine the association between the clinical signs and the motif of IBV strains, we determined the amino acids sequences at the cleavage sites. The deduced amino acids that were obtained from the nucleotide sequences at the cleavage site between the S1 and S2 proteins of each strain are shown in Table 1.

The “RRFRR” motif was the most common (30/61) and was found in strains other than the GVI-1 (JP-IV) and GI-3 (Gray) genotypes. However, in the GI-18 (JP-I) genotype, which has the largest number of examined strains, there were only two RRFRR strains, and the RRSRR motif, including various other motifs, was predominant. The second predominant was the “RRSRR” motif, which was more common in the GI-18 (JP-I) genotype, while the “HRRKR” motif was found only in the GVI-1 (JP-IV) genotype.

### 3.4. Amino Acids Related to the Receptor-Binding Domain (RBD)

It has been reported that the NTD of S1 (aa. 19–272, most N-terminal 253 aa of the mature S1) is both required and sufficient for binding to the respiratory tract [18]. Moreover, amino acids N38, H43, P63, and T69 of M41 S1 were critical to establishing binding within the domain. Therefore, these amino acids’ polymorphism (s) were compared among the genotypes. The results that are presented in Table 3 show that the amino acids at positions 38, 43, 63, and 69 were critical for binding to the trachea in terms of M41 spike attachment; however, the diversity at the positions that are mentioned above in these amino acids was also observed in Japanese IBVs.

Recently, the motifs “^26^YxYY^29^” and “^34^FxPPxxWxLH^43^” of high-affinity aminopeptidase N (APN) peptides in chickens have been identified in IBV S1-NTD [19]. APN may be one of the IBV functional receptors. When comparing these amino acid sequences in Japanese IBVs, the motif “^26^YxYY^29^” was conserved in all Japanese IBV strains. However, the motif “^34^FxPPxxWxLH^43^” was conserved in all the strains belonging to GI-3 and some strains belonging to GI-1, GI-18 and GVI-1 (Table 3). Specifically, in all GI-7 or 19 genotypes strains, the amino acid at position 43 was substituted from H to Q. However, this amino acid was the H that was described above in the M41 strain [19], not the nephropathogenic strain.

Nevertheless, QX-RBD amino acids 110 to 112 (KIP), considered important for binding to the kidney in the nephropathogenic QX strain, and classified into GI-19 [20], were not observed in Japanese IBVs, including the GI-19 (JP-III) genotype (Table 4).

### 3.5. Potential N-glycosylation Sites

N-glycosylation occurs at asparagine (N) residues in the amino acid motif asparagine-x-serine/threonine (N-X-S/T) [21]. Therefore, the loss or acquisition of N-glycosylation sites in the spike glycoprotein was predicted, including their positions on representative strains of each genotype (Table 5). Similarities were observed between the positions of the predicted N-glycosylation sites in each S1 glycoprotein of the different Japanese genotype strains, with nine sites shared across all the strains (Table 5). Among these sites, the three N-linked glycosylation sites (at positions 103, 163, and 237 at position in the M41 strain) were considered critically important in previous studies [21], and they were also all conserved in Japanese IBVs. However, their N-glycosylation sites differed among the genotypes. Specifically, although we observed 15 to 17 N-glycosylation sites in the S1 glycoprotein in the GI-1 (Mass) strains, as previously reported [14], 18 or 19 N-glycosylation sites were also present in the GI-19 (JP-III) strains [22]. However, while 19 or 20 N-glycosylation sites in the S1 glycoprotein were observed in the GI-7 (JP-II) strains, only 11 or 12 N-glycosylation sites were present in the GVI-1 (JP-IV) strains. Furthermore, the predicted N-glycosylation site at position 116 (unique to JP-II), and that at position 200, observed in the QX strain (genotype GI-19) [22], were not observed in the GI-19 (JP-III) genotype strains in Japan.

## 4. Discussion

The S1 glycoprotein determines the antigenicity and tissue tropism of IBVs [23], playing a vital role in inducing neutralizing antibodies and attachment to the host cell receptors [18]. Therefore, the genetic analysis of the complete S1 gene of IBV is critical.

To date, genotyping has been performed on Japanese strains using regions containing HVR-1 and 2 [11,12], but the analysis of the predicted S1 glycoprotein amino acid sequences was insufficient. Here, the complete S1 gene of 61 IBV strains, classified into seven genotypes (including ten vaccine strains which were attenuated strains that were derived from field viruses in Japan), was determined and analyzed.

First, the genotyping using the complete S1 gene was almost consistent with the Japanese typing that was based on partial nucleotide sequences, including HVR-1 and 2, with three exceptions. These strains (JP/Nagasaki/2013, JP/Kochi/2013, and JP/Nagasaki/2016) belonged to genotype JP-III in the classification using Japanese typing. However, they were classified into distinct genotypes from GI-13 and 19 when using the full S1 gene length, whereas most JP-III genotype strains were classified into the GI-19 genotype. Therefore, these three strains that were derived from the GI-19 and GI-13 genotypes were all considered recombinants. This result is consistent with that of a previous analysis [10], which showed that most of the recombination breakpoints of field IBV strains occur in the intermediate region between HVR1, HVR2, and HVR3. Recently, many such field IBV recombinants of this kind have been increasing worldwide [24]. For example, the recent Chinese isolate (CK/CH/SCMY/160315) was identified as a novel recombinant virus that was derived from the H120 (GI-1), 4/91 (GI-13), and LDT3-A (GI-28) live attenuated vaccine strains, and the LJL/08-1 (GI-19) field strain. Besides, in Japan, field strains that are potential recombinants have also been reported through the analysis of S1 and S2 [12]. However, little analysis of Japanese IBV recombinants using the complete genome has been reported. Therefore, it would be necessary to conduct a genetic analysis using the complete genome to clarify the detailed genetic background of domestic epidemic IBV strains.

By analyzing the S1 glycoprotein’s predicted amino acid sequence, seven types of S1 protein cleavage recognition motifs were observed among the Japanese IBV strains. However, no association was found between the clinical signs and the motif of the isolates. This finding was consistent with the cleavage recognition motif of the S1 gene being reportedly irrelevant to viral pathogenicity and tissue tropism [25].

In general, IBV infects ciliated epithelial cells in the respiratory tract of chickens. While some IBV strains replicate locally, others can disseminate to various organs, including the kidneys [20]. By conducting studies using the IBV M41 strain, the reference strain of the GI-1 (Mass) genotype, alpha-2,3-linked sialic acids were identified as IBV receptors [23]. Furthermore, it was shown that the amino acids at positions 38, 43, 63, and 69 were critical for binding to the trachea in terms of the M41 spike attachment. By comparing the IBV receptor-related amino acids that were detected using this M41 strain, various substitutions were observed among Japanese IBV genotypes. For example, a recent study on the QX strain, classified into the GI-19 genotype, reported that amino acids 110 to 112 (KIP) of the S1glycoprotein were sufficient to extend its tropism toward the kidney [20]. However, this KIP motif was not found in Japanese IBV strains, some isolated from nephritis, including the GI-19 (JP-III) genotype. Nevertheless, although the two motifs that were found in the recent APN study [19], the amino acid at position 43 of ^34^FxPPxxWxLH^43^ was substituted with Q instead of H, they were found in genotypes GI-7 (JP-II) and GI-19 (JP-III). Since IBV strains belonging to GI-7 and 19 are known to be the nephropathogenic type in foreign countries [8,16], it would be interesting to examine the effect of this amino acid substitution on the viral pathogenicity or cellular tropism.

Furthermore, studies have reported that the glycosylation of viral structural proteins influences many of the processes and outcomes of an IBV infection, including antigenicity, infectivity, and receptor-binding [14,26]. Therefore, comparing N-glycosylation sites among IBV strains is essential to examine such biological functions. Using bioinformatic techniques, previous analyses of IBV strains identified 17 sites in the S1 sequences of strains belonging to the Mass serotype. Additionally, they observed that the total number of predicted sites was different from that of the QX-like strains [27]. Previous studies have also suggested that some N-linked glycosylation sites were critically important for virus replication and infectivity [21]. Furthermore, these sites were all conserved in Japanese IBVs (Table 5), which is functionally important. For example, N to D/Q mutations in N212 and N276, abolished the infectivity of the recombinant IBV [21]. However, the loss or gain of N-glycosylation sites differed among the genotypes. Interestingly, the JP-II (GI-7) genotype was composed mainly nephritis-derived strains and contained more numbers (20–21) than those that were predicted in the S1 glycoprotein. Therefore, since the mechanism of nephritis that is caused by IBV in chickens remains unknown, it was of particular interest to analyze the virus’ nephropathogenicity and the effect of these glycosylations.

In conclusion, we have shown the phylogenetic relationship between Japanese IBV strains and foreign strains in this study. Additionally, by analyzing the amino acid polymorphism of the S1 glycoprotein among Japanese genotypes, a diversity was observed based on the genotype-specific amino acid residue and the position of the potential N-glycosylation sites. This finding is significant since the glycosylation of viral structural proteins has been shown to influence many of the processes and outcomes of IBV infections, including antigenicity, infectivity, and receptor-binding. Thus, to unequivocally determine the contributions of such amino acid residues to the virus’ pathogenicity, it is imperative to generate viruses with specific mutations using reverse genetics [28,29]. This study’s obtained findings would be useful for elucidating IBV’s pathogenicity and improving vaccine development strategies.

## Figures and Tables

**Table 1:** Japanese IBV strains employed in this study.

Strain	Isolation Year	Length (bp)	Clinical Signs	Genotype Based on HVR-1,2	Genotype Based on Complete Sequence of S1 Gene	Cleavage Site
JP/KH/64	1964	1632	Respiratory	JP-I	GI-18	RRSRR
JP/Shizuoka/71	1971	1632	Respiratory	JP-I	GI-18	RRSRR
JP/Chiba/74	1974	1632	Respiratory	JP-I	GI-18	RRSRR
JP/Chiba/77	1977	1632	Egg drop	JP-I	GI-18	RRSRR
JP/Chiba/80	1980	1632	Respiratory	JP-I	GI-18	RRSRR
JP/Mie/92	1992	1629	Nephritis	JP-I	GI-18	HRFRR
JP/Akita/92	1992	1632	Respiratory	JP-I	GI-18	RRFKR
JP/Nagano/95	1995	1629	Nephritis	JP-I	GI-18	RRSKR
JP/Yamanashi/95	1995	1632	Respiratory	JP-I	GI-18	RRSRR
JP/Shizuoka/98	1998	1626	Nephritis	JP-I	GI-18	RRSRR
JP/Chiba/98	1998	1632	Nephritis	JP-I	GI-18	RRFKR
JP/Toyama/2000	2000	1632	Nephritis	JP-I	GI-18	HRFRR
JP/Okayama-1/2000	2000	1632	Nephritis	JP-I	GI-18	RRFKR
JP/Okayama-2/2000	2000	1632	Nephritis	JP-I	GI-18	RRFKR
JP/Ibaraki/2003	2003	1632	Nephritis	JP-I	GI-18	RRSKR
JP/Ehime/2003	2003	1632	Depression, respiratory	JP-I	GI-18	RRFKR
JP/Kagoshima/2008	2008	1629	Depression, respiratory	JP-I	GI-18	RRSRR
JP/Kagoshima-1/2014	2014	1632	Nephritis	JP-I	GI-18	RRFRR
JP/Yamagata/2017	2017	1629	Rise in mortality	JP-I	GI-18	RRSRR
JP/Kumamoto/2019	2019	1632	Nephritis	JP-I	GI-18	RRFRR
C-78		1629	Vaccine strain	JP-I	GI-18	RRSRR
GN		1632	Vaccine strain	JP-I	GI-18	RRFKR
S95		1632	Vaccine strain	JP-I	GI-18	RRSKR
JP/Miyazaki/89	1989	1614	Nephritis	JP-II	GI-7	RRFRR
JP/Yamanashi/93	1993	1614	Nephritis	JP-II	GI-7	RRFRR
JP/Osaka/2000	2000	1614	Nephritis	JP-II	GI-7	RRFRR
JP/Kanagawa/2001	2001	1614	Nephritis	JP-II	GI-7	RRSKR
JP/Yamagata/2011	2011	1614	Nephritis	JP-II	GI-7	RRFKR
JP/Nagasaki/2015	2015	1614	Respiratory	JP-II	GI-7	RRFRR
Miyazaki		1614	Vaccine strain	JP-II	GI-7	RRFRR
TM86		1614	Vaccine strain	JP-II	GI-7	RRFRR
JP/Shimane/98	1998	1617	Respiratory	JP-III	GI-19	HRFRR
JP/Aichi/2000	2000	1620	Nephritis	JP-III	GI-19	RRFRR
JP/Fukui/2000	2000	1620	Respiratory	JP-III	GI-19	RRFRR
JP/Shimane/2002	2002	1614	Nephritis	JP-III	GI-19	RRFRR
JP/Okayama-5/2004	2004	1611	Egg drop	JP-III	GI-19	RRFRR
JP/Chiba/2004	2004	1617	Nephritis	JP-III	GI-19	RRFRR
JP/Wakayama-13/2006	2006	1620	Rise in mortality	JP-III	GI-19	RRFRR
JP/Saitama/2007	2007	1617	Egg drop	JP-III	GI-19	RRFRR
JP/Kagoshima-1/2009	2009	1620	Depression, diarrhea	JP-III	GI-19	RRFRR
JP/Kagoshima-2/2009	2009	1620	Egg drop	JP-III	GI-19	RRFRR
JP/Kochi/2013	2013	1617	Rise in mortality	JP-III	Recombinant	RRFRR
JP/Nagasaki/2013	2013	1620	Depression, diarrhea	JP-III	Recombinant	RRFRR
JP/Gifu/2015	2015	1620	Egg drop	JP-III	GI-19	RRFRR
JP/Nagasaki/2016	2016	1617	Respiratory, diarrhea	JP-III	Recombinant	RRFKR
JP/Chiba/2018	2018	1620	Respiratory	JP-III	GI-19	RRFRR
AK01		1620	Vaccine strain	JP-III	GI-19	RRFRR
JP/Ibaraki/168-1/2009	2009	1638	Egg drop	JP-IV	GVI-1	HRRKR
JP/Chiba/2010	2010	1638	Nephritis	JP-IV	GVI-1	HRRKR
JP/Kagoshima-3/2014	2014	1638	Respiratory	JP-IV	GVI-1	HRRKR
JP/Ishida/51	1951	1611	Respiratory	Mass	GI-1	RRFRR
JP/Nerima/53	1953	1611	Respiratory	Mass	GI-1	RRFRR
JP/Kagoshima-2/2014	2014	1611	Nephritis	Mass	GI-1	RRFRR
Nerima		1611	Vaccine strain	Mass	GI-1	RRFRR
Kita-1		1605	Vaccine strain	Mass	GI-1	RRFRR
KU		1611	Vaccine strain	Mass	GI-1	RRFRR
JP/Kagoshima-4/2014	2014	1623	Rise in mortality	Gray	GI-3	RRSRR
ON		1629	Vaccine strain	Gray	GI-3	RRSRR
JP/Wakayama/2003	2003	1617	Depression, diarrhea	4/91	GI-13	RRFRR
JP/Okayama-7/2004	2004	1617	Nephritis	4/91	GI-13	RRSRR
JP/Saitama/2006	2006	1617	Respiratory	4/91	GI-13	RRFRR

**Table 2:** List of RT-PCR primers.

Name		Sequence (5ʹ-3ʹ)	Position ^a^	Length (bp)	Reference
Forward	Reverse				
15F		AGGAATGGTAAGTTRCTRGTWAGAG	20343–20367	671	Mase et al., 2004 [11]
	26Rm	GCGCAGTACCRTTRAYAAAATAAGC	21013–20989		Mase et al., 2004 [11]
19F		GCAGTGTTTGTTACGCATTG	20689–20708	1333	in this study
	1R	CATAACTAACATAAGGGCAA	22021–22002		in this study

^a^ Position is given for the S1 gene of strain Beadette42 (Acc.No.NC001451).

**Table 3:** Amino acids based on their relationship to the receptor-binding domain.

Genotype	Amino Acid Position of Amino Acids at the S1 Glycoprotein											
	26	28	29	34	36	37	38	40	42	43	63	69
Mass (GI-1)	Y	Y	Y	F	P	P	D/N	W	L	H/Q	P/S	T/I
Gray (GI-3)	Y	Y	Y	F	P	P	N	W	L	H	S	A
JP-I GI-18)	Y	Y	Y	F/L/Y	P	P/S/G	L/F/P/S/V	W	L/V/I	H	S/H/A	A
JP-II (GI-7)	Y	Y	Y	F	P	P	D/N	W	L	Q	P/L/R	S
JP-III (GI-19)	Y	Y	Y	F	P	P/S	D/N/T/E	W	L	Q	P/S/N/T/A/Q	V
JP-IV (GVI-1)	Y	Y	Y	F	P	P	L/S	W	L	H/Y	G/H	A
4/91 (GI-13)	Y	Y	Y	F	P	G	P/Q	W	L	H/Y	P/S	T

**Table 4:** Amino acids related to the kidney binding.

Genotype	Corresponding Position of Amino Acids at S1 Glycoprotein		
	110	111	112
Ck/SWE/0658946/10 (QX)	K	I	P
Mass (GI-1)	M/V	L/V/I	Q
Gray (GI-3)	F/I	L	P
JP-I (GI-18)	L/F/R/S	I	Q/N/A/E
JP-II (GI-7)	F	V	P
JP-III (GI-19)	M/Q/L	I	P/K
JP-IV (GVI-1)	K/I	L	D/K/G
4/91 (GI-13)	M	I	P

**Table 5:** Potential N-glycosylation sites of Japanese IBV genotypes.

Strain	Genotype	Amino Acid Position at POTENTIAL N-glycosylation ^a^																										Number of Glycosylation Site
M41(AY851295)	Mass (GI-1)	51	-	77	-	103 ^b^	-	-	144	163	178	-	-	212	237	247	264	271	276	-	306	-	425	447	-	513	530	17
JP/Ishida/51(LC662594)	Mass (GI-1)	51	-	77	-	103	-	-	144	163	178	-	-	212	237	247	264	271	276	-	306	-	425	447	-	513	530	17
JP/Nerima/53(LC662595)	Mass (GI-1)	51	-	-	-	103	-	-	144	163	178	-	-	212	237	247	264	-	276	-	306	-	425	447	-	513	530	15
Gray(L14069)	GI-3	51	-	75	-	103	-	-	150	169	184	-	-	218	243	253	270	277	282	-	312	-	431	453	-	519	536	17
JP/Kagoshima-4/2014(LC662600)	Gray (GI-3)	-	-	75	-	103	-	-	148	167	182	-	-	216	241	251	268	275	280	-	310	-	429	451	-	517	534	16
QX(MN548289)	QX (GI-19)	52	55	76	92	104	117	141	147	166	181	-	200	215	240	250	267	274	279	282	309	405	428	450	457	516	533	21
JP/Shimane/98(LC662575)	JP-III (GI-19)	51	54	-	-	103	-	140	146	165	180	-	-	214	239	249	266	273	278	281	308	-	427	449	-	515	532	19
JP/Aichi/2000(LC662576)	JP-III (GI-19)	52	55	-	-	104	-	141	147	166	181	-	-	215	240	250	267	274	279	282	309	-	428	450	-	516	-	18
JP/Fukui/2000(LC662577)	JP-III (GI-19)	52	55	76	-	104	-	141	147	166	181	-	-	215	240	250	267	274	279	-	309	405	428	450	-	516	-	19
JP/Shimane/2002(LC662578)	JP-III (GI-19)	50	53	74	-	102	-	139	145	164	179	-	-	213	238	248	265	272	277	280	307	-	426	448	-	514	-	19
JP/Kagoshima-1/2009(LC662583)	JP-III (GI-19)	52	-	76	-	104	-	141	147	166	181	-	-	215	240	250	267	274	279	282	309	-	428	450	-	516	-	18
4/91(KF377577)		-	54	75	-	103	-	-	146	165	180	-	-	214	239	249	266	273	278	281	308	-	427	449	456	515	533	19
JP/Wakayama/2003(LC662602)	4/91 (GI-13)	-	54	75	-	103	-	-	146	165	180	-	-	214	239	249	266	273	278	-	308	-	427	449	456	515	532	18
JP/Okayama-7/2004(LC662603)	4/91 (GI-13)	-	54	75	-	103	-	-	146	165	180	184	-	214	239	249	266	273	278	281	308	-	427	449	456	515	532	20
JP8127(AY296744)	GI-18	51	-	75	-	103	-	-	151	170	185	-	-	219	244	254	271	278	283	-	314	-	433	455	-	521	538	17
JP/KH/64(LC634083)	JP-I (GI-18)	51	-	75	-	103	-	-	151	170	185	-	-	219	244	254	271	278	283	-	313	-	432	454	-	520	-	16
JP/Akita/92(LC662550)	JP-I (GI-18)	51	-	75	-	103	-	-	151	170	185	-	-	219	244	254	271	278	283	-	313	-	432	454	-	520	537	17
JP/Nagano/95(LC662551)	JP-I (GI-18)	51	-	75	91	103	-	-	151	170	185	-	-	219	244	254	271	278	283	-	313	-	431	453	-	519	536	18
JP/Kagoshima-1/2014(LC662561)	JP-I (GI-18)	51	-	75	-	103	-	-	151	170	185	-	-	219	244	254	271	278	283	-	313	-	432	454	461	520	537	18
TP/64(AY606320)	GI-7	51	-	77	-	103	116	139	145	164	179	-	-	213	238	248	265	272	277	-	308	-	427	449	-	515	532	19
JP/Miyazaki/89(LC662567)	JP-II (GI-7)	51	-	77	-	103	116	139	145	164	179	-	-	213	238	248	265	272	277	280	307	-	426	448	-	514	531	20
JP/Nagasaki/2015(LC662572)	JP-II (GI-7)	51	-	-	-	103	116	139	145	164	179	-	-	213	238	248	265	272	277	280	307	-	426	448	-	514	531	19
TC07-2(GQ265948)	GVI-1	-	55	75	-	104	-	-	148	167	182	-	-	216	241	256	-	275	280	-	314	411	-	456	-	522	539	16
JP/Ibaraki/168-1/2009(LC662591)	JP-IV (GVI-1)	-	55	75	-	104	-	-	148	167	182	-	-	216	241	256	-	275	280	-	314	-	-	456	-	522	539	15
JP/Kagoshima-3/2014(LC662593)	JP-IV (GVI-1)	-	55	75	-	104	-	-	148	167	-	-	-	216	241	256	-	275	280	-	314	-	-	456	-	522	539	14

^a^ The numbers indicate the amino acid positions of each strain. ^b^ Bold italics indicate positions that are suggested to be functionally important [19]. -: deletion.

## Data Availability

All data generated and analyzed during this study are included in this article.

## References

[1] Jackwood M., de Wit J.J., Swayne D.E., Boulianne M., Logue C.M., McDougald L.R., Nair V., Suarez D.L. (2020). Infectious bronchitis virus. Diseases of Poultry.

[2] De Wit J.J., Cook J.K.A. (2019). Spotlight on avian pathology: Infectious bronchitis virus. Avian Pathol..

[3] Jordan B. (2017). Vaccination against infectious bronchitis virus: A continuous challenge. Vet. Microbiol..

[4] Cavanagh D. (2007). Coronavirus avian infectious bronchitis virus. Vet. Res..

[5] Koch G., Hartog L., Kant A., Van Roozelaar D.J. (1990). Antigenic domains on the peplomer protein of avian infectious bronchitis virus: Correlation with biological functions. J. Gen. Virol..

[6] Kant A., Koch G., Van Roozelaar D.J., Kusters J.G., Poelwijk F.A.J., Van der Zeijst B.A.M. (1992). Location of antigenic sites defined by neutralizing monoclonal antibodies on the S1 avian infectious bronchitis virus glycopolypeptide. J. Gen. Virol..

[7] Lee E.K., Jeon W.J., Lee Y.J., Jeong O.M., Choi J.G., Kwon J.H., Choi K.S. (2008). Genetic diversity of avian infectious bronchitis virus isolates in Korea between 2003 and 2006. Avian Dis..

[8] Ren M., Zhang L., Hou Y., Zhao Y., Han Z., Sun J., Liu S. (2020). Genetic, antigenic, and pathogenic characteristics of infectious bronchitis virus GI-7/TW-II in China. Avian Dis..

[9] Wang C.H., Huang Y.C. (2000). Relationship between serotypes and genotypes based on the hypervariable region of the S1 gene of infectious bronchitis virus. Arch. Virol..

[10] Valastro V., Holmes E.C., Britton P., Fusaro A., Jackwood M.W., Cattoli G., Monne I. (2016). S1 gene-based phylogeny of infectious bronchitis virus: An attempt to harmonize virus classification. Infect. Genet. Evol..

[11] Mase M., Tsukamoto K., Imai K., Yamaguchi S. (2004). Phylogenetic analysis of avian infectious bronchitis virus strains isolated in Japan. Arch. Virol..

[12] Mase M., Gotou M., Inoue D., Watanabe S., Iseki H. (2021). Genotyping of infectious bronchitis viruses isolated in Japan during 2008−2019. J. Vet. Med Sci..

[13] Shang J., Zheng Y., Yang Y., Liu C., Geng Q., Luo C., Zhang W., Li F. (2018). Cryo-EM structure of infectious bronchitis coronavirus spike protein reveals structural and functional evolution of coronavirus spike proteins. PLoS Pathog..

[14] Parsons L.M., Bouwman K.M., Azurmendi H., De Vries R.P., Cipollo J.F., Verheije M.H. (2019). Glycosylation of the viral attachment protein of avian coronavirus is essential for host cell and receptor binding. J. Biol. Chem..

[15] Kumar S., Stecher G., Tamura K. (2016). MEGA7: Molecular Evolutionary Genetics Analysis Version 7.0 for Bigger Datasets. Mol. Biol. Evol..

[16] Lim T.H., Lee H.J., Lee D.H., Lee Y.N., Park J.K., Youn H.N., Kim M.S., Lee J.B., Park S.Y., Choi I.S. (2011). An emerging recombinant cluster of nephropathogenic strains of avian infectious bronchitis virus in Korea. Infect. Genet. Evol..

[17] Pohuang T., Chansiripornchai N., Tawatsin A., Sasipreeyajan J. (2011). Sequence analysis of S1 genes of infectious bronchitis virus isolated in Thailand during 2008-2009: Identification of natural recombination in the field isolates. Virus Genes.

[18] Promkuntod N., van Eijndhoven R.E.W., de Vrieze G., Gröne A., Verheije M.H. (2014). Mapping of the receptor-binding domain and amino acids critical for attachment in the spike protein of avian coronavirus infectious bronchitis virus. Virology.

[19] Sun X., Li L., Pan L., Wang Z., Chen H., Shao C., Yu J., Ren Y., Wang X., Huang X. (2021). Infectious bronchitis virus: Identification of Gallus gallus APN high-affinity ligands with antiviral effects. Antivir. Res..

[20] Bouwman K.M., Parsons L.M., Berends A.J., de Vries R.P., Cipollo J.F., Verheije M.H. (2020). Three Amino Acid Changes in Avian Coronavirus Spike Protein Allow Binding to Kidney Tissue. J. Virol..

[21] Zheng J., Yamada Y., Fung T.S., Huang M., Chia R., Liu D.X. (2018). Identification of N-linked glycosylation sites in the spike protein and their functional impact on the replication and infectivity of coronavirus infectious bronchitis virus in cell culture. Virology.

[22] Stevenson-Leggett P., Armstrong S., Keep S., Britton P., Bickerton E. (2021). Analysis of the avian coronavirus spike protein reveals heterogeneity in the glycans present. J. Gen. Virol..

[23] Wickramasinghe I.N.A., de Vries R.P., Gröne A., de Haan C.A.M., Verheije M.H. (2011). Binding of Avian Coronavirus Spike Proteins to Host Factors Reflects Virus Tropism and Pathogenicity. J. Virol..

[24] Jiang Y., Cheng X., Zhao X., Yu Y., Gao M., Zhou S. (2020). Recombinant infectious bronchitis coronavirus H120 with the spike protein S1 gene of the nephropathogenic IBYZ strain remains attenuated but induces protective immunity. Vaccine.

[25] Jackwood M.W., Hilt D.A., Callison S.A., Lee C.W., Plaza H., Wade E. (2001). Spike glycoprotein cleavage recognition site analysis of infectious bronchitis virus. Avian Dis..

[26] Zhang X., Deng T., Lu J., Zhao P., Chen L., Qian M., Guo Y., Qiao H., Xu Y., Wang Y. (2020). Molecular characterization of variant infectious bronchitis virus in China, 2019: Implications for control programmes. Transbound. Emerg. Dis..

[27] Abro S.H., Ullman K., Belák S., Baule C. (2012). Bioinformatics and evolutionary insight on the spike glycoprotein gene of QX-like and Massachusetts strains of infectious bronchitis virus. Virol. J..

[28] Casais R., Thiel V., Siddell S.G., Cavanagh D., Britton P. (2001). Reverse genetics system for the avian coronavirus infectious bronchitis virus. J. Virol..

[29] Van Beurden S.J., Berends A.J., Krämer-Kühl A., Spekreijse D., Chénard G., Philipp H.C., Mundt E., Rottier P.J.M., Verheije M.H. (2017). A reverse genetics system for avian coronavirus infectious bronchitis virus based on targeted RNA recombination. Virol. J..

