# Correction: Mase et al. Genetic Analysis of the Complete S1 Gene in Japanese Infectious Bronchitis Virus Strains. *Viruses* 2022, *14*, 716

**DOI:** 10.3390/v14102098

**Published:** 2022-09-22

**Authors:** Masaji Mase, Kanae Hiramatsu, Satoko Watanabe, Hiroshi Iseki

**Affiliations:** 1 National Institute of Animal Health, National Agriculture and Food Research Organization, 3-1-5 Kannondai, Tsukuba 305-0856, Japan; 2 United Graduate School of Veterinary Sciences, Gifu University, 1-1 Yanagido, Gifu 501-1193, Japan; 3 Graduate School of Life and Environmental Sciences, Osaka Prefecture University, Izumisano 598-8531, Japan; 4 Oita Livestock Hygiene Service Center of Oita Prefecture, 442 Onozuru, Oita 870-1153, Japan

## Error in Table

In the original publication [1], there was a mistake in “**Table 2. List of RT-PCR primers**” as published. **The primers in Table 2 are not for infectious bronchitis virus, and the sequence of avian reovirus amplification is mistakenly listed**. The corrected Table 2 appears below. The authors state that the scientific conclusions are unaffected. This correction was approved by the Academic Editor. The original publication has also been updated.

## Figures and Tables

**Table 2:** List of RT-PCR primers.

Name		Sequence (5’-3’)	Position ^a^	Length (bp)	Reference
Forward	Reverse				
15F		AGGAATGGTAAGTTRCTRGTWAGAG	20343–20367	671	Mase et al., 2004 [11]
	26Rm	GCGCAGTACCRTTRAYAAAATAAGC	21013–20989		Mase et al., 2004 [11]
19F		GCAGTGTTTGTTACGCATTG	20689–20708	1333	in this study
	1R	CATAACTAACATAAGGGCAA	22021–22002		in this study

^a^ Position is given for the S1 gene of strain Beadette42 (Acc.No.NC001451).
